# Higher Serum Melatonin Levels during the First Week of Malignant Middle Cerebral Artery Infarction in Non-Surviving Patients

**DOI:** 10.3390/brainsci9120346

**Published:** 2019-11-28

**Authors:** Leonardo Lorente, María M. Martín, Pedro Abreu-González, Rafael Sabatel, Luis Ramos, Mónica Argueso, Jordi Solé-Violán, Juan J. Cáceres, Alejandro Jiménez, Victor García-Marín

**Affiliations:** 1 Intensive Care Unit, Hospital Universitario de Canarias, Ofra s/n, 38320 La Laguna, Santa Cruz de Tenerife, Spain; 2 Intensive Care Unit, Hospital Universitario Nuestra Señora de Candelaria, Crta del Rosario s/n, 38010 Santa Cruz de Tenerife, Spain; mar.martinvelasco@gmail.com; 3 Department of Physiology, Faculty of Medicine, University of the La Laguna, Ofra s/n, 38320 La Laguna, Santa Cruz de Tenerife, Spain; pabreu@ull.es; 4 Department of Radiology, Hospital Universitario de Canarias, Ofra s/n, 38320 La Laguna, Santa Cruz de Tenerife, Spain; rsabatel@gmail.com; 5 Intensive Care Unit, Hospital General La Palma, Buenavista de Arriba s/n, 38713 La Palma, Breña Alta, Spain; lramosgomez@gmail.com; 6 Intensive Care Unit, Hospital Clínico Universitario de Valencia, Avda. Blasco Ibáñez nº17-19, 46004 Valencia, Spain; moni_begasa@hotmail.com; 7 Intensive Care Unit, Hospital Universitario Dr. Negrín, CIBERES, Barranco de la Ballena s/n, 35010 Las Palmas de Gran Canaria, Spain; jsolvio@gobiernodecanarias.org; 8 Intensive Care Unit, Hospital Insular, Plaza Dr. Pasteur s/n, 35016 Las Palmas de Gran Canaria, Spain; juanjose.caceresagra@gobiernodecanarias.org; 9 Research Unit, Hospital Universitario de Canarias, Ofra s/n, 38320 La Laguna, Santa Cruz de Tenerife, Spain; ajimenezsosa@gmail.com; 10 Department of Neurosurgery, Hospital Universitario de Canarias, Ofra s/n., 38320 La Laguna, Santa Cruz de Tenerife, Spain; vicgarmar666@gmail.com

**Keywords:** melatonin, malignant middle cerebral artery infarction, patients, mortality, prognosis

## Abstract

Objective: The activation of different physiopathological pathways (neuroinflammation, apoptosis, and oxidation) can lead to secondary brain injury in ischemic stroke, and in animal models the administration of melatonin has reduced that secondary injury. Lower levels of serum melatonin were found at the time of admission of cerebral infarction in surviving patients than in non-surviving patients. Thus, we carried out this prospective and observational study with the aim of exploring serum melatonin levels in the first week of a malignant middle cerebral artery infarction (MMCAI) in surviving and non-surviving patients, and to explore the capacity of those levels to predict mortality. Methods: Patients with severe MMCAI, defined as computed tomography showing acute infarction in more than 50% of the territory and Glasgow Coma Scale (GCS) lower than 9, were included in the study. We measured serum melatonin concentrations at days 1, 4, and 8 of MMCAI. Mortality at 30 days was the endpoint of our study. Results: Non-surviving patients (*n* = 34) compared to surviving patients (*n* = 34) showed higher serum melatonin levels at days 1 (*p* < 0.001), 4 (*p* < 0.001), and 8 (*p* = 0.001) of MMCAI. Serum melatonin concentrations at days 1, 4, and 8 of MMCAI had an area under the curve (AUC) (95% confidence interval (CI)) in the prediction of mortality of 0.89 (0.80–0.96; *p* < 0.001), 0.81 (0.68–0.91; *p* < 0.001), and 0.82 (0.68–0.92; *p* < 0.001), respectively. Conclusions: The novel findings of our study were that serum melatonin levels in the first week of MMCAI were higher in non-surviving patients, and were able to predict mortality.

## 1. Introduction

Many deaths and disabilities occur due to ischemic stroke [1]. After a primary brain injury, the activation of different physiopathological pathways (neuroinflammation, apoptosis, and oxidation) can lead to secondary brain injury in ischemic stroke [2,3,4,5,6]. A reduction of secondary brain injury in ischemic stroke has been found in animal models with the administration of melatonin, due to a lower development of neuroinflammation and oxidation [2,3,4,5,6]. Melatonin reduces neuroinflammation by decreasing proinflammatory cytokines (such as interleukin-6 and tumor necrosis factor-α) and by increasing anti-inflammatory cytokines (such as interleukin-10). Melatonin reduces oxidation by increasing several antioxidant enzymes (such as glutathione reductase and glutathione peroxidase) and by its potent capacity as scavenger of reactive oxygen species.

Scarce data exist about blood melatonin levels in patients with ischemic stroke [7,8,9]. Higher serum melatonin levels have been found in healthy controls than in patients with ischemic stroke [7,8]. Our team found lower serum melatonin levels at the time of admission of cerebral infarction in surviving patients than in non-surviving patients [9]. In addition, our team also found higher serum melatonin levels in non-surviving than in surviving patients at admission of traumatic brain injury (TBI) [10] and of spontaneous intracerebral hemorrhage (ICH) [11]. We believe that our findings in patients with cerebral infarction, TBI, and ICH showing higher melatonin levels in non-surviving than in surviving patients could be due to the efforts of patients to reduce the great neuroinflammation and oxidation states. We hypothesized that non-surviving patients with cerebral infarction would have higher serum melatonin levels in the first week of cerebral infarction than surviving patients. Therefore, we carried out this study with the objectives of exploring serum melatonin levels in the first week of a malignant middle cerebral artery infarction (MMCAI) in surviving and non-surviving patients, and to explore the capacity of those levels to predict mortality. 

## 2. Materials and Methods

### 2.1. Design and Subjects

Six Spanish hospitals participated in this observational and prospective study, and the Institutional Board of each hospital approved the protocol study, namely, H. Universitario Dr. Negrín (Las Palmas de Gran Canaria), H. Universitario Nuestra Señora de Candelaria (Santa Cruz de Tenerife), H. Clínico Universitario de Valencia, H. General de La Palma, H. Insular (Las Palmas de Gran Canaria), and H. Universitario de Canarias (La Laguna). A relative of each patient signed the written informed consent to include the patients in the study. 

Patients with severe malignant middle cerebral artery infarction (MMCAI), defined as computed tomography showing acute infarction in more than of 50% of the territory and Glasgow Coma Scale (GCS) [12] lower than 9 were included in the study. Patients with inflammatory disease, less than 18 years of age, or with malignant disease were excluded from the study.

The following variables were collected at the moment of MMCAI diagnosis: sex, arterial hypertension, diabetes mellitus, and age. We also recorded CGS, thrombolysis, volumen infarction, midline shift, hemorrhagic transformation, lactic acid, bilirubin, sodium, glycemia, creatinine, pressure of arterial oxygen (PaO_2_), fraction inspired of oxygen (FI0_2_), platelets, hemoglobin, leukocytes, international normalized ratio (INR), fibrinogen, activated partial thromboplastin time (aPTT), Acute Physiology and Chronic Health Evaluation II (APACHE II) score [13], and decompressive craniectomy. Thirty-day mortality was the endpoint of our study.

Regarding the treatment of patients, according to the American guidelines published in 2007 [14], intravenous thrombolysis therapy was not used in patients in whom CT showed an extent infarction (hypodensity in at least 1/3 of the cerebral hemisphere). Thrombectomy was not used because the improvement in outcomes was unclear. Instead, decompressive surgery was used for malignant edema according to the neurosurgeon’s criteria.

### 2.2. Determinations of Serum Melatonin Levels 

We froze serum samples at −80 °C on days 1, 4, and 8 of MMCAI. Previously, we determined serum levels of melatonin in 50 patients [9] and of malondialdehyde in 64 patients [15] on day 1 of MMCAI. Subsequently, with the inclusion of more patients, we determined serum malondialdehyde concentrations at days 1, 4, and 8 of MMCAI in 68 patients [16]. In this study, we determined serum melatonin concentrations at days 1, 4, and 8 in those 68 MMCAI patients to determine its mortality prognostic capability and its association with malondialdehyde concentrations (as a biomarker of lipid peroxidation) [17,18].

Serum melatonin concentrations were determined by the ELISA method using a kit from Immuno Biological Laboratories (IBL Hamburg GmbH, Hamburg, Germany) and a microplate spectrophotometer reader (Benchmark Plus, Bio-Rad, Hercules, CA, USA). The detection limit was 0.13 pg/mL, the inter-assay variation coefficient was 11.1%, and the intra-assay variation coefficient was 6.4%. 

### 2.3. Statistical Methods

We used medians (percentiles 25th and 75th) and frequencies (percentages) to describe continuous and categorical variables, respectively. We assessed the normality using the Kolmogorov–Smirnov test and serum melatonin levels were not adjusted to the normal distribution. We used the Wilcoxon–Mann–Whitney test and the chi-squared test to compare continuous and categorical variables between both patient groups (30-day survivors and non-survivors), respectively. We carried out receiver operating characteristic (ROC) analyses to test the capacity of serum melatonin levels at days 1, 4, and 8 of MMCAI to predict mortality, and we reported area under the curve (AUC) and 95% confidence interval (CI) for those levels at each day. In addition, we reported specificity, sensitivity, positive predictive value, positive likelihood ratio, negative predictive value, and negative likelihood ratio for the cut-offs (selected using the Youden J index) of serum melatonin levels at days 1, 4, and 8 of MMCAI. We constructed Kaplan–Meier 30-day mortality curves with patients showing higher and lower serum melatonin levels than >2.93 pg/mL (cut-off value selected by the Youden J index). We carried out a multiple logistic regression analysis to determine the association between serum melatonin levels and other variables with 30-day mortality. Statistical analyses were carried out using LogXact 4.1 (Cytel Co., Cambridge, MA, USA), NCSS 2000 (Kaysville, UT, USA), and SPSS 17.0 (IBM, Chicago, IL, USA), and *p*-value cut-off <0.05 was used to establish significant differences.

## 3. Results

We did not find significant differences between non-surviving (*n* = 34) and surviving MMCAI patients (*n* = 34) in terms of sex, arterial hypertension, diabetes mellitus, age, thrombolysis, infarction volume, midline shift, hemorrhagic transformation, lactic acid, bilirubin, sodium, glycemia, creatinine, PaO_2_/FI0_2_ ratio, hemoglobin, leukocytes, INR, fibrinogen, aPTT, APACHE- II score, and decompressive craniectomy (Table 1). Non-surviving MMCAI patients compared to surviving patients showed lower GCS (*p* = 0.01), lower platelet count (*p* = 0.02), and higher serum melatonin levels at admission (*p* < 0.001) (Table 1). No patients underwent endovascular thrombectomy, 16 patients underwent decompressive craniectomy, and 21 patients underwent intravenous thrombolysis. No significant differences were found in the 30-day survival rate in patients with decompressive craniectomy (56%, 9 of 16 patients) or without it (48%, 25 of 52 patients) (*p* = 0.78), nor in patients with intravenous thrombolysis (52%, 11 of 21 patients) or without it (50%, 23 of 47 patients) (*p* = 0.99).

**Figure 1:**
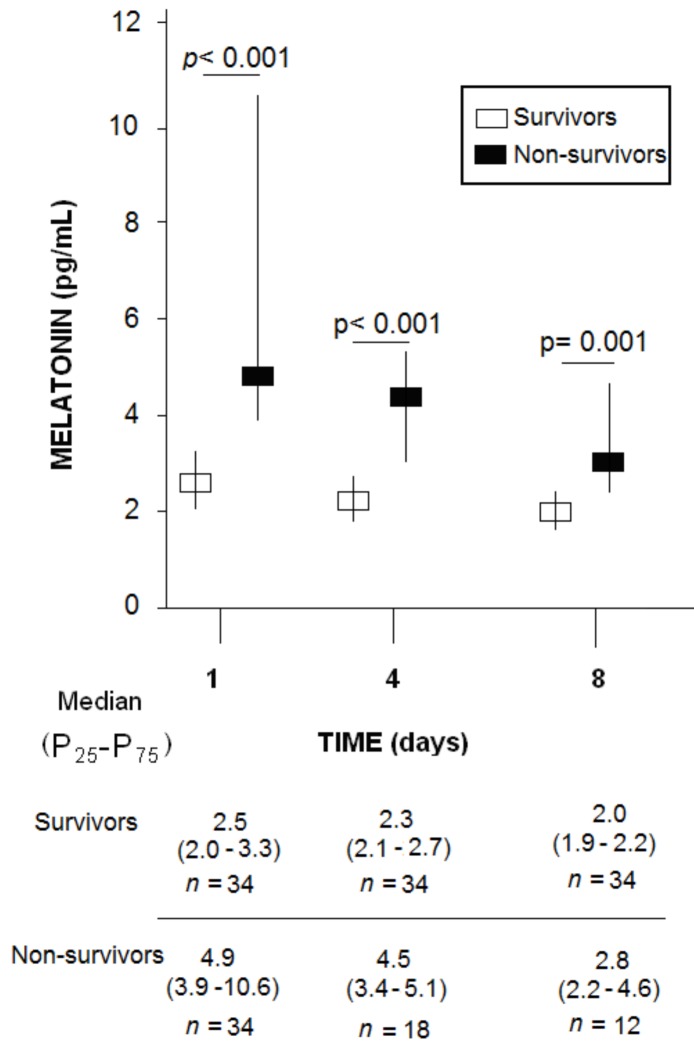
Serum melatonin levels at days 1, 4, and 8 of malignant middle cerebral artery infarction in 30-day surviving and non-surviving patients.

In addition, non-surviving patients also showed higher serum melatonin levels at days 4 (*p* < 0.001) and 8 (*p* = 0.001) of MMCAI (Figure 1). We did not find significant differences in serum melatonin levels at day 1 between the 16 patients who died by day 4 and the 18 patients who were still alive on day 4 (*p* = 0.48), nor in serum melatonin levels at day 4 between the 6 patients alive at day 4 who died by day 8 and the 12 patients who were still alive on day 8 (*p* = 0.49). We did not find significant differences in serum melatonin levels between patients who underwent decompressive craniectomy at days 1 (*p* = 0.47), 4 (*p* = 0.29), and 8 (*p* = 0.10), nor in serum melatonin levels between patients who underwent intravenous thrombolysis at days 1 (*p* = 0.32), 4 (*p* = 0.55), and 8 (*p* = 0.87).

Serum melatonin concentrations at days 1, 4, and 8 of MMCAI had an AUC (95% CI) in the prediction of mortality of 0.89 (0.80–0.96; *p* < 0.001), 0.81 (0.68–0.91; *p* < 0.001), and 0.82 (0.68–0.92; *p* < 0.001), respectively. Table 2 showed specificity, sensitivity, positive predictive value, positive likelihood ratio, negative predictive value, and negative likelihood ratio of serum melatonin levels cut-offs at days 1, 4, and 8 of MMCAI.

**Figure 2:**
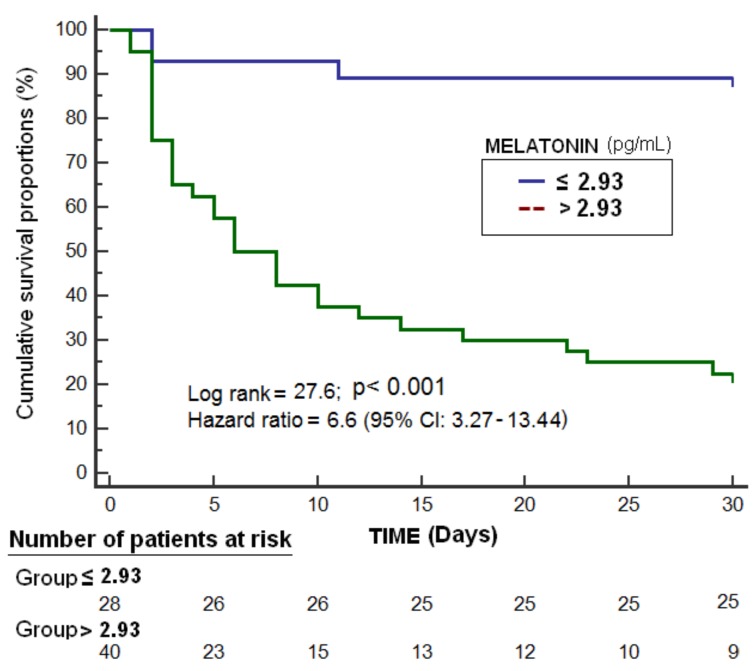
Survival curves at 30 days using serum melatonin levels lower than or equal to vs. higher than 2.93 pg/mL.

Kaplan–Meier analysis showed that patients with serum melatonin levels >2.93 pg/mL had a higher 30-day mortality rate (hazard ratio = 6.6; 95% CI = 3.27–13.44; *p* < 0.001) (Figure 2). Logistic regression analysis showed that serum melatonin levels were associated with 30-day mortality after control for lactic acid, GCS, and platelet count (odds ratio = 2.369; 95% CI = 1.328−4.227; *p* = 0.004) (Table 3). 

We found a positive association between serum concentrations of melatonin and malondialdehyde at days 1 (rho = 0.38; *p* = 0.002), 4 (rho = 0.44; *p* = 0.001), and 8 (rho = 0.63; *p* < 0.001) of MMCAI.

## 4. Discussion 

The novel findings of our study were that serum melatonin levels in the first week of MMCAI were higher in non-surviving patients, and were able to predict mortality. In a previous study carried out by our team, we determined serum melatonin levels on admission of MMCAI and we found higher serum melatonin levels in non-surviving patients [9]. Thus, the novel aspects of our current study are that serum melatonin levels also at days 4 and 8 of MMCAI were higher in non-surviving than in surviving patients, and at days 4 and 8 of MMCAI were also able to predict mortality. We think the clinician would find it interesting that taking a blood biomarker at any moment of the first week of MMCAI could help in the prediction of mortality. 

Previously, we found higher malondialdehyde levels at day 1 of MMCAI [15] and during the first week of MMCAI [16] in non-surviving than in surviving patients. The positive association between serum concentrations of malondialdehyde (as a biomarker of lipid peroxidation) and melatonin during the first week of MMCAI is another interesting new finding of our study. 

Since melatonin levels have been shown to be higher in healthy patients compared to ischemic patients [7,8], one would predict that melatonin in non-survivors would be lower than in surviving MMCAI patients. However, we found higher serum melatonin levels in non-surviving than in surviving patients, and a positive association between serum levels of melatonin and malondialdehyde. Similar findings have been found by our team in patients with severe traumatic brain injury [10] or spontaneous intracerebral hemorrhage [11]. We believe that those high serum melatonin levels in non-surviving patients could represent a response to the great neuroinflammation (not assessed in our study) and oxidation states (assessed in our study by serum malondialdehyde concentrations). However, those high bloodstream melatonin levels in non-surviving patients are not enough to compensate this unbalanced clinical state and to avoid the eventual death of the patient.

The administration of melatonin in animal models with cerebral ischemia has shown the beneficial effects of reduced neuroinflammation, apoptosis, oxidation, and brain edema leading to lower secondary brain injury [19,20,21,22,23,24,25,26,27,28,29,30,31,32,33]. In a randomized controlled trial, the use of oral melatonin was studied during three days before carotid endarterectomy. Blood samples were taken at baseline, pre-anesthesia, carotid reconstruction finished, and 6, 24, and 72 hours after carotid endarterectomy. The patient group receiving melatonin compared to the patient group receiving placebo showed decreased expression of proinflammatory cytokines (tumor necrosis factor-α and interleukin-6) and increased expression of antioxidant molecules (superoxide dismutase and glutathione peroxidase) [34]. However, the use of melatonin in the acute phase of cerebral infarction has not been reported in humans.

We would like to acknowledge some limitations in our study, such as the fact that we did not determine serum melatonin levels in patients with mild or moderate MMCAI, in other critically ill patients, or in healthy subjects. In addition, we did not measure serum levels of interleukins to assess neuroinflammation. Nor did we measure serum levels of interleukins in healthy subjects to compare with MMCAI patients. Finally, in the American guidelines published in 2007 [14], it was established that intravenous thrombolysis therapy could be used in patients in whom CT did not show a multilobar infarction (hypodensity 1/3 cerebral hemisphere), that the usefulness of thrombectomy in improving outcomes after stroke was unclear, and that decompressive surgery for malignant edema might be recommended for treatment of seriously affected patients. In guidelines published in 2018 [1], it was established that intravenous thrombolysis therapy could be used in patients without imaging evidence of ischemic injury involving more than one third of the middle cerebral artery (MCA) territory. Regarding thrombectomy, it should be used in patients meeting all the following criteria: pre-stroke modified Rankin scale (mRS) ≤1, occlusion of the internal carotid artery or MCA segment 1, age ≥18 years, National Institutes of Health Stroke Scale (NIHSS) ≥6, Alberta Stroke Program Early Computed Tomography Score (ASPECTS) ≥6, and treatment initiated (groin puncture) within 6 hours of symptom onset. In addition, regarding decompressive craniectomy, it could be used in patients with unilateral MCA infarctions who deteriorate neurologically within 48 hours despite medical therapy, especially in patients ≤60 years of age. In our series, no patients underwent endovascular thrombectomy, and we did not find significant differences in the survival rate at 30 days in patients with or without decompressive craniectomy, nor in patients with or without intravenous thrombolysis. However, we want to think that the beneficial effects reported with the melatonin use in animal models of cerebral ischemia and the potential use of melatonin levels to predict mortality of MMACI patients found in our study will stimulate research on the role of blood melatonin levels to predict cerebral infarction prognosis.

## 5. Conclusions

The novel findings of our study were that serum melatonin levels in the first week of MMCAI were higher in non-surviving patients and were able to predict mortality. 

## Figures and Tables

**Table 1:** Clinical and biochemical characteristics of 30-day survivor and non-survivor malignant middle cerebral artery infarction patients.

	Non-Survivors (*n* = 34)	Survivors (*n* = 34)	*p*-Value
Gender female-*n* (%)	13 (38.2)	14 (41.2)	0.99
Arterial hypertension-*n* (%)	16 (47.1)	19 (55.9)	0.63
Diabetes mellitus-*n* (%)	9 (26.5)	4 (11.8)	0.22
Age (years)-m (p 25–75)	63 (53–70)	59 (47–68)	0.36
GCS score-m (p 25–75)	6 (3–7)	7 (6–8)	0.01
Thrombolysis-*n* (%)	10 (29.4)	11 (32.4)	0.99
Volumen infarction (mL)-m (p 25–75)	180 (60–277)	173 (100–231)	0.64
Midline shift (mm)-m (p 25–75)	9.0 (3.5–15.0)	6.0 (2.5–11.5)	0.43
Hemorrhagic transformation-*n* (%)	6 (17.6)	7 (20.6)	0.99
Lactic acid (mmol/L)-m (p 25–75)	1.55 (1.00–2.70)	1.20 (0.90–1.70)	0.05
Sodium (mEq/L)-m (p 25–75)	140 (139–145)	139 (136–145)	0.38
Bilirubin (mg/dL)-m (p 25–75)	0.60 (0.33–1.10)	0.60 (0.40–0.83)	0.95
Creatinine (mg/dL)-m (p 25–75)	1.00 (0.70–1.25)	0.80 (0.60–1.13)	0.19
Glycemia (g/dL)-m (p 25–75)	136 (118–162)	127 (100–170)	0.40
PaO_2_ (mmHg)-m (p 25–75)	115 (94–267)	156 (105–293)	0.26
PaO_2_/FI0_2_ ratio-m (p 25–75)	254 (192–325)	300 (198–369)	0.24
Platelets-m×10^3^/mm^3^ (p 25–75)	175 (136–216)	202 (171–265)	0.02
Leukocytes-m×10^3^/mm^3^ (p 25–75)	13.9 (9.7–20.1)	12.4 (9.6–16.9)	0.32
Hemoglobin (g/dL)-m (p 25-75)	12.5 (11.0–14.8)	12.1 (11.4–14.0)	0.81
Fibrinogen (mg/dL)-m (p 25–75)	419 (337–631)	443 (416–489)	0.90
INR-m (p 25–75)	1.20 (1.01–1.31)	1.06 (1.00–1.20)	0.07
aPTT (seconds)-m (p 25–75)	27 (26–32)	28 (25–30)	0.91
APACHE-II score-m (p 25–75)	22 (19–27)	20 (16–25)	0.06
Decompressive craniectomy-*n* (%)	7 (20.6)	9 (26.5)	0.78
Melatonin (pg/mL)-m (p 25–75)	4.9 (3.9–10.6)	2.5 (2.0–3.3)	<0.001

GCS, Glasgow Coma Scale; *n*, sample size; m, median; p 25–75, percentile 25th–75th; PaO_2_, pressure of arterial oxygen; FIO_2_, fraction inspired oxygen; INR, international normalized ratio; aPTT, activated partial thromboplastin time; APACHE II, Acute Physiology and Chronic Health Evaluation.

**Table 2:** Thirty-day mortality prognostic capability of serum melatonin levels at days 1, 4, and 8 of malignant middle cerebral artery infarction.

	Day 1	Day 4	Day 8
Cut-off of melatonin (pg/mL)	>2.93	>3.14	>2.27
Sensitivity (95% CI)	91% (76%–98%)	83% (59%–96%)	75% (43%–95%)
Specificity (95% CI)	74% (56%–87%)	88% (73%–97%)	82% (66%–93%)
Positive predictive value (95% CI)	78% (66%–86%)	79% (59%–91%)	60% (40%–77%)
Positive likelihood ratio (95% CI)	3.4 (1.9–6.1)	7.1 (2.8–18.2)	4.3 (1.9–9.4)
Negative predictive value (95% CI)	89% (74%–96%)	91% (78%–97%)	90% (78%–96%)
Negative likelihood ratio (95% CI)	0.12 (0.04–0.40)	0.19 (0.07–0.50)	0.30 (0.10–0.80)

CI, confidence interval.

**Table 3:** Logistic regression analysis to predict 30-day mortality.

Variable	Odds Ratio	95% Confidence Interval	*p*-Value
Serum melatonin levels (pg/mL)	2.369	1.328–4.227	0.004
Glasgow Coma Scale (points)	0.695	0.472–1.022	0.06
Lactic acid (mmol/L)	1.134	0.576–2.232	0.72
Platelet count (each 1000/mm^3^)	0.997	0.987–1.008	0.63
